# 3,6-Bis(4-chloro­phen­yl)-*N* ^1^,*N* ^4^-bis­(1-phenyl­eth­yl)-1,2,4,5-tetra­zine-1,4-di­carboxamide

**DOI:** 10.1107/S1600536812003765

**Published:** 2012-02-04

**Authors:** Na-Bo Sun, Jia-Bin Ni, Guo-Wu Rao

**Affiliations:** a College of Biology and Environmental Engineering, Zhejiang Shuren University, Hangzhou 310015, People’s Republic of China; b College of Pharmaceutical Science, Zhejiang University of Technology, Hangzhou 310014, People’s Republic of China

## Abstract

In the title mol­ecule, C_32_H_28_Cl_2_N_6_O_2_, the amide-substituted N atoms of the tetra­zine ring deviate from the approximate plane of the four other atoms in the ring by 0.468 (3) and 0.484 (3) Å, forming a boat conformation. The dihedral angle between the two phenyl rings is 67.0 (1)° and that between the two chloro-substituted benzene rings is 73.8 (1)°. Two intra­molecular N—H⋯N hydrogen bonds are observed.

## Related literature

For chemical reactions of 1,2,4,5-tetra­zine derivatives, see: Domingo *et al.* (2009 ▶); Lorincz *et al.* (2010 ▶). For their biological activities, see: Devaraj *et al.* (2009 ▶); Eremeev *et al.* (1978 ▶, 1980 ▶); Han *et al.* (2010 ▶); Neunhoeffer (1984 ▶); Sauer (1996 ▶). For anti-tumor activity of 1,2,4,5-tetra­zine derivatives, see: Hu *et al.* (2002 ▶, 2004 ▶); Rao & Hu (2005 ▶, 2006 ▶). For standard bond lengths, see: Allen *et al.* (1987 ▶). For the synthesis of the title compound, see: Abdel-rahman *et al.* (1968 ▶); Hu *et al.* (2004 ▶); Rao & Hu (2006 ▶).
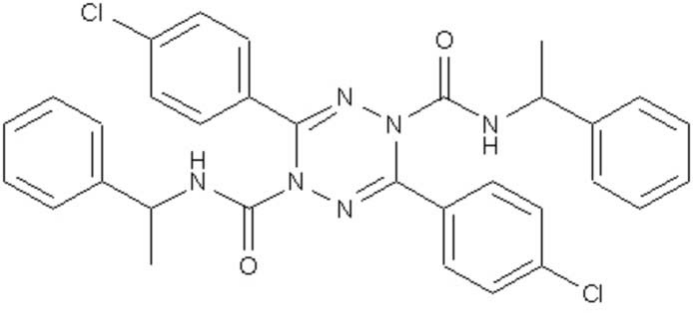


## Experimental

### 

#### Crystal data

C_32_H_28_Cl_2_N_6_O_2_
*M* *_r_* = 599.50
Orthorhombic, 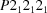

*a* = 9.715 (2) Å
*b* = 14.725 (3) Å
*c* = 21.159 (5) Å
*V* = 3027.0 (12) Å^3^
*Z* = 4
Mo *K*α radiation
μ = 0.25 mm^−1^
*T* = 298 K
0.23 × 0.19 × 0.12 mm

#### Data collection

Bruker SMART CCD diffractometer
Absorption correction: multi-scan (*SADABS*; Bruker, 1997 ▶) *T*_min_ = 0.944, *T*_max_ = 0.970
19445 measured reflections
7314 independent reflections
4680 reflections with *I* > 2σ(*I*)
*R*_int_ = 0.033

#### Refinement

*R*[*F* ^2^ > 2σ(*F* ^2^)] = 0.042
*wR*(*F* ^2^) = 0.099
*S* = 1.01
7314 reflections
380 parameters
H-atom parameters constrained
Δρ_max_ = 0.13 e Å^−3^
Δρ_min_ = −0.15 e Å^−3^
Absolute structure: Flack (1983 ▶), with 3185 Friedel pairs
Flack parameter: 0.03 (5)

### 

Data collection: *SMART* (Bruker, 1997 ▶); cell refinement: *SAINT* (Bruker, 1997 ▶); data reduction: *SAINT*; program(s) used to solve structure: *SHELXS97* (Sheldrick, 2008 ▶); program(s) used to refine structure: *SHELXL97* (Sheldrick, 2008 ▶); molecular graphics: *ORTEP-3 for Windows* (Farrugia, 1997 ▶); software used to prepare material for publication: *WinGX* (Farrugia, 1999 ▶).

## Supplementary Material

Crystal structure: contains datablock(s) I, global. DOI: 10.1107/S1600536812003765/lh5409sup1.cif

Supplementary material file. DOI: 10.1107/S1600536812003765/lh5409Isup2.cdx

Structure factors: contains datablock(s) I. DOI: 10.1107/S1600536812003765/lh5409Isup3.hkl

Supplementary material file. DOI: 10.1107/S1600536812003765/lh5409Isup4.cml

Additional supplementary materials:  crystallographic information; 3D view; checkCIF report

## Figures and Tables

**Table 1:** Hydrogen-bond geometry (Å, °)

*D*—H⋯*A*	*D*—H	H⋯*A*	*D*⋯*A*	*D*—H⋯*A*
N3—H3⋯N2	0.86	2.21	2.613 (2)	109
N6—H6⋯N5	0.86	2.13	2.573 (2)	112

**Fig. 1.**
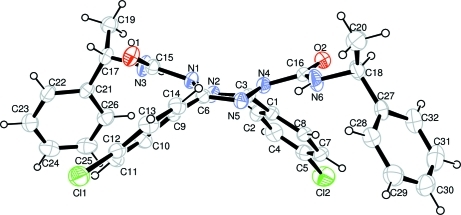
The molecular structure of (I), shown with 30% probability displacement ellipsoids.

**Fig. 2.**
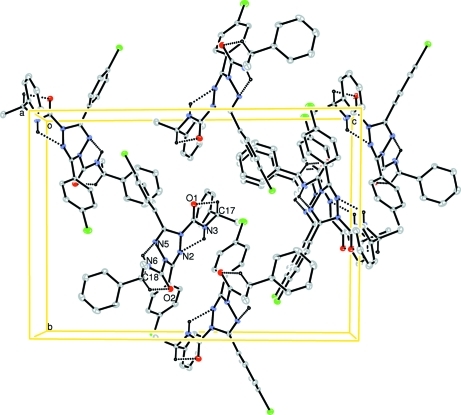
A portion of the crystal packing of (I). Hydrogen bonds are shown as dashed lines. H atoms not involved in hydrogen bonding were omitted for clarity.

## supplementary crystallographic information

### Comment

Tetrazine derivatives have high activity in chemical reactions (Domingo *et al.*, 2009; Lorincz *et al.*, 2010), and have been widely used in medicines and pesticides (Devaraj *et al.*, 2009; Eremeev *et al.*, 1978, 1980; Han *et al.*, 2010; Neunhoeffer, 1984; Sauer, 1996). In a continuation of our studies of antitumor activities in 1,2,4,5-tetrazine derivatives (Hu *et al.*, 2002, 2004; Rao & Hu, 2005, 2006), we have obtained a yellow crystalline compound, (I). However, IR, NMR, and MS studies failed to prove whether the substituted groups of the nitrogen are located at the 1,4 or 1,2 position. The structure was confirmed by single-crystal X-ray diffraction. The molecular structure of (I) is illustrated in Fig. 1.

The N2═C3 [1.282 (2) Å] and N5═C6 [1.285 (2) Å] bonds are typical as are the C3—N4 [1.407 (3) Å], N4—N5 [1.429 (2) Å], C6—N1 [1.396 (2) Å] and N1—N2 [1.417 (2) Å] bonds (Allen *et al.*, 1987). The tetrazine ring is a 1,4-dihydro structure with the N-substituted groups at the 1,4-positions.

In (I), atoms N2, C3, N5 and C6 are approximately planar, with the largest deviation from this plane being 0.0236 (9) Å. Atoms N1 and N4 deviate from this plane by 0.468 (3) and 0.484 (3) Å, respectively. The dihedral angle between the N2/C3/N5/C6 plane and the N1/N2/C6 plane is 38.00 (17)°, and between the N2/C3/N5/C6 plane and the N4/N5/C3 plane is 38.81 (14)°. The tetrazine ring has a boat conformation. The dihedral angles between the N2/C3/N5/C6 plane and the two benzene rings at the 3,6-positions are 35.91 (10) and 42.87 (8)°, respectively. And the two benzene rings form a dihedral angle of 73.8 (1)°. Two intramolecular N—H···N hydrogen bonds are observed.

### Experimental

The title compound was prepared according to the procedure of Abdel-rahman *et al.* (1968); Hu *et al.* (2004); Rao & Hu, (2006). A solution of the compound in ethanol was concentrated gradually at room temperature to afford yellow blocks.

### Refinement

H atoms were included in calculated positions and refined using a riding model. H atoms were given isotropic displacement parameters equal to 1.2 (or 1.5 for methyl H atoms) times the equivalent isotropic displacement parameters of their parent atoms, and C—H distances were set to 0.96 Å for methyl H atoms, 0.93 Å for phenyl H atoms and 0.98 Å for methine H atoms, while N—H distances were set to 0.86 Å.

### Crystal data

**Table:**

C_32_H_28_Cl_2_N_6_O_2_	*F*(000) = 1248
*M**_r_* = 599.50	*D*_x_ = 1.316 Mg m^−^^3^
Orthorhombic, *P*2_1_2_1_2_1_	Mo *K*α radiation, λ = 0.71073 Å
Hall symbol: P 2ac 2ab	Cell parameters from 4171 reflections
*a* = 9.715 (2) Å	θ = 2.5–21.5°
*b* = 14.725 (3) Å	µ = 0.25 mm^−^^1^
*c* = 21.159 (5) Å	*T* = 298 K
*V* = 3027.0 (12) Å^3^	Block, yellow
*Z* = 4	0.23 × 0.19 × 0.12 mm

### Data collection

**Table:**

Bruker SMART CCD diffractometer	7314 independent reflections
Radiation source: fine-focus sealed tube	4680 reflections with *I* > 2σ(*I*)
Graphite monochromator	*R*_int_ = 0.033
φ and ω scans	θ_max_ = 28.2°, θ_min_ = 1.7°
Absorption correction: multi-scan (*SADABS*; Bruker, 1997)	*h* = −12→12
*T*_min_ = 0.944, *T*_max_ = 0.970	*k* = −19→17
19445 measured reflections	*l* = −27→23

### Refinement

**Table:**

Refinement on *F*^2^	Hydrogen site location: inferred from neighbouring sites
Least-squares matrix: full	H-atom parameters constrained
*R*[*F*^2^ > 2σ(*F*^2^)] = 0.042	*w* = 1/[σ^2^(*F*_o_^2^) + (0.0431*P*)^2^] where *P* = (*F*_o_^2^ + 2*F*_c_^2^)/3
*wR*(*F*^2^) = 0.099	(Δ/σ)_max_ < 0.001
*S* = 1.01	Δρ_max_ = 0.13 e Å^−^^3^
7314 reflections	Δρ_min_ = −0.15 e Å^−^^3^
380 parameters	Extinction correction: *SHELXL97* (Sheldrick, 2008), Fc^*^=kFc[1+0.001xFc^2^λ^3^/sin(2θ)]^-1/4^
0 restraints	Extinction coefficient: 0.0032 (5)
Primary atom site location: structure-invariant direct methods	Absolute structure: Flack (1983), with 3185 Friedel pairs
Secondary atom site location: difference Fourier map	Flack parameter: 0.03 (5)

### Special details

**Table:**

Geometry. All e.s.d.'s (except the e.s.d. in the dihedral angle between two l.s. planes) are estimated using the full covariance matrix. The cell e.s.d.'s are taken into account individually in the estimation of e.s.d.'s in distances, angles and torsion angles; correlations between e.s.d.'s in cell parameters are only used when they are defined by crystal symmetry. An approximate (isotropic) treatment of cell e.s.d.'s is used for estimating e.s.d.'s involving l.s. planes.
Refinement. Refinement of *F*^2^ against ALL reflections. The weighted *R*-factor *wR* and goodness of fit *S* are based on *F*^2^, conventional *R*-factors *R* are based on *F*, with *F* set to zero for negative *F*^2^. The threshold expression of *F*^2^ > σ(*F*^2^) is used only for calculating *R*-factors(gt) *etc*. and is not relevant to the choice of reflections for refinement. *R*-factors based on *F*^2^ are statistically about twice as large as those based on *F*, and *R*-factors based on ALL data will be even larger.

### Fractional atomic coordinates and isotropic or equivalent isotropic displacement parameters (Å^2^)

**Table:**

	*x*	*y*	*z*	*U*_iso_*/*U*_eq_	
Cl1	0.97748 (8)	0.65714 (4)	0.24050 (3)	0.0762 (2)	
Cl2	1.29647 (10)	1.51972 (5)	0.14950 (5)	0.1031 (3)	
O1	1.00558 (16)	0.89564 (10)	0.01136 (7)	0.0586 (4)	
N2	1.06405 (18)	1.12227 (11)	0.05805 (8)	0.0481 (4)	
C9	0.9347 (2)	0.92381 (13)	0.13933 (9)	0.0413 (4)	
N1	0.99625 (16)	1.03724 (10)	0.05423 (7)	0.0456 (4)	
C12	0.9598 (2)	0.76006 (14)	0.20118 (10)	0.0504 (5)	
N4	0.85399 (17)	1.15911 (11)	0.10225 (8)	0.0458 (4)	
C14	0.8196 (2)	0.87854 (15)	0.16048 (10)	0.0499 (5)	
H14	0.7329	0.9036	0.1539	0.060*	
N5	0.84906 (17)	1.07490 (11)	0.13578 (8)	0.0470 (4)	
C6	0.92133 (19)	1.01363 (13)	0.10807 (9)	0.0422 (5)	
C3	0.9867 (2)	1.18387 (13)	0.08194 (9)	0.0443 (5)	
C15	1.0540 (2)	0.97121 (14)	0.01349 (9)	0.0464 (5)	
C13	0.8316 (2)	0.79653 (15)	0.19127 (11)	0.0542 (6)	
H13	0.7535	0.7661	0.2052	0.065*	
O2	0.73843 (16)	1.29115 (10)	0.08359 (8)	0.0599 (4)	
C16	0.7439 (2)	1.21948 (15)	0.11254 (10)	0.0496 (5)	
C11	1.0759 (2)	0.80464 (16)	0.18125 (11)	0.0594 (6)	
H11	1.1625	0.7800	0.1886	0.071*	
C21	1.3807 (2)	0.92538 (15)	−0.03766 (10)	0.0486 (5)	
C10	1.0625 (2)	0.88628 (15)	0.15023 (11)	0.0536 (6)	
H10	1.1409	0.9166	0.1364	0.064*	
C2	1.1644 (2)	1.29943 (15)	0.05976 (11)	0.0595 (6)	
H2	1.1902	1.2655	0.0246	0.071*	
C1	1.0499 (2)	1.27347 (13)	0.09516 (9)	0.0467 (5)	
C7	1.0867 (3)	1.40218 (15)	0.16293 (11)	0.0608 (6)	
H7	1.0604	1.4374	0.1973	0.073*	
C22	1.4427 (3)	0.84197 (17)	−0.04630 (11)	0.0667 (7)	
H22	1.3939	0.7953	−0.0657	0.080*	
C27	0.5366 (2)	1.26970 (15)	0.23883 (11)	0.0546 (6)	
C8	1.0115 (2)	1.32642 (13)	0.14618 (10)	0.0515 (5)	
H8	0.9339	1.3108	0.1695	0.062*	
N3	1.15792 (19)	1.00173 (12)	−0.02169 (9)	0.0609 (5)	
H3	1.1793	1.0584	−0.0200	0.073*	
C28	0.5385 (2)	1.20923 (16)	0.28849 (12)	0.0659 (7)	
H28	0.5316	1.1474	0.2800	0.079*	
N6	0.6510 (2)	1.18814 (14)	0.15343 (10)	0.0700 (6)	
H6	0.6655	1.1360	0.1705	0.084*	
C25	1.5885 (3)	0.9750 (2)	0.01361 (13)	0.0771 (8)	
H25	1.6371	1.0200	0.0349	0.093*	
C26	1.4546 (3)	0.99204 (16)	−0.00713 (12)	0.0654 (6)	
H26	1.4150	1.0487	−0.0003	0.078*	
C18	0.5259 (2)	1.23737 (17)	0.17096 (12)	0.0665 (7)	
H18	0.5190	1.2910	0.1438	0.080*	
C17	1.2367 (2)	0.94135 (14)	−0.06312 (10)	0.0515 (5)	
H17	1.1894	0.8826	−0.0643	0.062*	
C19	1.2379 (3)	0.97911 (17)	−0.12969 (12)	0.0685 (7)	
H19A	1.1450	0.9886	−0.1437	0.103*	
H19B	1.2829	0.9369	−0.1574	0.103*	
H19C	1.2866	1.0358	−0.1302	0.103*	
C5	1.2012 (3)	1.42479 (15)	0.12797 (13)	0.0647 (7)	
C24	1.6486 (3)	0.8930 (2)	0.00299 (13)	0.0769 (8)	
H24	1.7387	0.8823	0.0159	0.092*	
C31	0.5579 (3)	1.3885 (2)	0.3163 (2)	0.0925 (10)	
H31	0.5638	1.4501	0.3257	0.111*	
C23	1.5758 (3)	0.8270 (2)	−0.02661 (13)	0.0766 (8)	
H23	1.6164	0.7708	−0.0337	0.092*	
C4	1.2394 (3)	1.37479 (17)	0.07648 (13)	0.0691 (7)	
H4	1.3159	1.3917	0.0528	0.083*	
C29	0.5502 (3)	1.2375 (2)	0.35034 (14)	0.0809 (8)	
H29	0.5510	1.1951	0.3829	0.097*	
C32	0.5464 (3)	1.36083 (17)	0.25330 (15)	0.0758 (8)	
H32	0.5453	1.4039	0.2211	0.091*	
C20	0.3998 (3)	1.1790 (2)	0.15957 (15)	0.0967 (10)	
H20A	0.3969	1.1610	0.1160	0.145*	
H20B	0.3185	1.2131	0.1696	0.145*	
H20C	0.4042	1.1260	0.1859	0.145*	
C30	0.5606 (3)	1.3271 (3)	0.36354 (15)	0.0865 (9)	
H30	0.5695	1.3464	0.4052	0.104*	

### Atomic displacement parameters (Å^2^)

**Table:**

	*U*^11^	*U*^22^	*U*^33^	*U*^12^	*U*^13^	*U*^23^
Cl1	0.1065 (5)	0.0540 (3)	0.0681 (4)	−0.0008 (4)	−0.0168 (4)	0.0070 (3)
Cl2	0.1104 (6)	0.0705 (5)	0.1283 (7)	−0.0315 (4)	−0.0333 (5)	−0.0013 (4)
O1	0.0634 (9)	0.0497 (8)	0.0625 (10)	−0.0088 (8)	0.0160 (8)	−0.0093 (7)
N2	0.0526 (10)	0.0440 (9)	0.0476 (10)	−0.0026 (9)	0.0097 (8)	−0.0061 (8)
C9	0.0368 (11)	0.0460 (11)	0.0410 (11)	0.0013 (9)	0.0029 (9)	−0.0042 (9)
N1	0.0480 (9)	0.0437 (9)	0.0450 (9)	−0.0011 (8)	0.0108 (8)	−0.0067 (7)
C12	0.0618 (15)	0.0495 (11)	0.0400 (12)	−0.0001 (11)	−0.0077 (11)	−0.0031 (9)
N4	0.0481 (10)	0.0448 (9)	0.0445 (10)	0.0062 (8)	0.0053 (8)	−0.0007 (8)
C14	0.0379 (11)	0.0606 (13)	0.0512 (13)	0.0000 (10)	0.0023 (10)	0.0009 (11)
N5	0.0456 (10)	0.0499 (10)	0.0454 (10)	0.0041 (8)	0.0043 (8)	−0.0036 (8)
C6	0.0336 (10)	0.0502 (12)	0.0429 (11)	0.0003 (9)	0.0001 (9)	−0.0064 (9)
C3	0.0518 (12)	0.0451 (11)	0.0360 (10)	0.0044 (10)	0.0010 (9)	−0.0016 (9)
C15	0.0469 (12)	0.0487 (12)	0.0436 (12)	0.0032 (10)	0.0036 (10)	−0.0060 (9)
C13	0.0486 (13)	0.0627 (14)	0.0512 (13)	−0.0106 (11)	0.0032 (11)	0.0041 (11)
O2	0.0657 (10)	0.0547 (9)	0.0594 (10)	0.0113 (8)	−0.0077 (8)	−0.0019 (8)
C16	0.0518 (13)	0.0567 (13)	0.0404 (12)	0.0115 (11)	−0.0057 (10)	−0.0122 (10)
C11	0.0486 (13)	0.0668 (15)	0.0627 (15)	0.0100 (12)	−0.0052 (11)	0.0035 (12)
C21	0.0541 (13)	0.0506 (13)	0.0411 (12)	−0.0066 (11)	0.0122 (10)	−0.0053 (10)
C10	0.0368 (12)	0.0618 (13)	0.0622 (15)	−0.0017 (11)	0.0019 (10)	0.0066 (11)
C2	0.0738 (16)	0.0514 (13)	0.0533 (14)	−0.0008 (12)	0.0101 (12)	0.0003 (11)
C1	0.0554 (13)	0.0428 (11)	0.0418 (11)	0.0040 (10)	−0.0035 (10)	0.0010 (9)
C7	0.0797 (18)	0.0516 (14)	0.0510 (14)	0.0029 (13)	−0.0144 (13)	−0.0077 (10)
C22	0.0734 (17)	0.0642 (15)	0.0624 (15)	0.0048 (14)	0.0004 (13)	−0.0212 (12)
C27	0.0377 (12)	0.0559 (13)	0.0701 (15)	0.0103 (10)	0.0135 (11)	0.0005 (11)
C8	0.0596 (13)	0.0488 (12)	0.0461 (12)	0.0036 (11)	−0.0039 (10)	−0.0023 (10)
N3	0.0633 (12)	0.0470 (10)	0.0723 (13)	−0.0071 (9)	0.0300 (10)	−0.0172 (9)
C28	0.0610 (16)	0.0641 (15)	0.0725 (17)	0.0094 (13)	0.0017 (13)	0.0014 (13)
N6	0.0674 (13)	0.0775 (14)	0.0650 (13)	0.0332 (11)	0.0242 (10)	0.0116 (11)
C25	0.0812 (19)	0.0749 (19)	0.0752 (19)	−0.0292 (16)	−0.0165 (15)	0.0101 (15)
C26	0.0781 (17)	0.0501 (13)	0.0680 (16)	−0.0139 (13)	−0.0007 (14)	−0.0001 (11)
C18	0.0570 (14)	0.0773 (16)	0.0650 (16)	0.0278 (13)	0.0072 (12)	0.0059 (12)
C17	0.0533 (13)	0.0464 (12)	0.0548 (14)	−0.0051 (10)	0.0149 (11)	−0.0131 (10)
C19	0.0650 (16)	0.0838 (17)	0.0566 (16)	0.0082 (14)	0.0041 (12)	−0.0089 (13)
C5	0.0740 (17)	0.0475 (13)	0.0727 (18)	−0.0043 (12)	−0.0169 (14)	0.0068 (13)
C24	0.0664 (17)	0.100 (2)	0.0646 (18)	−0.0030 (17)	−0.0029 (14)	0.0123 (16)
C31	0.0721 (19)	0.0754 (19)	0.130 (3)	0.0021 (16)	0.014 (2)	−0.036 (2)
C23	0.0709 (18)	0.0835 (19)	0.0755 (18)	0.0180 (16)	−0.0007 (14)	−0.0117 (15)
C4	0.0699 (16)	0.0597 (15)	0.0776 (19)	−0.0072 (13)	0.0082 (14)	0.0135 (13)
C29	0.0678 (17)	0.102 (2)	0.0729 (19)	0.0139 (17)	0.0053 (15)	0.0014 (17)
C32	0.0687 (17)	0.0626 (16)	0.096 (2)	0.0111 (13)	0.0218 (16)	0.0081 (15)
C20	0.0718 (18)	0.127 (3)	0.091 (2)	0.0209 (19)	−0.0122 (16)	−0.032 (2)
C30	0.0590 (16)	0.119 (3)	0.082 (2)	0.0147 (19)	0.0102 (15)	−0.026 (2)

### Geometric parameters (Å, º)

**Table:**

Cl1—C12	1.737 (2)	C22—H22	0.9300
Cl2—C5	1.737 (3)	C27—C28	1.378 (3)
O1—C15	1.209 (2)	C27—C32	1.380 (3)
N2—C3	1.282 (2)	C27—C18	1.516 (3)
N2—N1	1.417 (2)	C8—H8	0.9300
C9—C14	1.376 (3)	N3—C17	1.465 (3)
C9—C10	1.379 (3)	N3—H3	0.8600
C9—C6	1.485 (3)	C28—C29	1.378 (4)
N1—C6	1.396 (2)	C28—H28	0.9300
N1—C15	1.415 (2)	N6—C18	1.463 (3)
C12—C11	1.371 (3)	N6—H6	0.8600
C12—C13	1.373 (3)	C25—C24	1.359 (4)
N4—C3	1.407 (3)	C25—C26	1.396 (4)
N4—C16	1.408 (3)	C25—H25	0.9300
N4—N5	1.429 (2)	C26—H26	0.9300
C14—C13	1.377 (3)	C18—C20	1.516 (4)
C14—H14	0.9300	C18—H18	0.9800
N5—C6	1.285 (2)	C17—C19	1.514 (3)
C3—C1	1.482 (3)	C17—H17	0.9800
C15—N3	1.332 (3)	C19—H19A	0.9600
C13—H13	0.9300	C19—H19B	0.9600
O2—C16	1.221 (2)	C19—H19C	0.9600
C16—N6	1.332 (3)	C5—C4	1.366 (4)
C11—C10	1.376 (3)	C24—C23	1.355 (4)
C11—H11	0.9300	C24—H24	0.9300
C21—C26	1.377 (3)	C31—C30	1.348 (4)
C21—C22	1.380 (3)	C31—C32	1.399 (4)
C21—C17	1.517 (3)	C31—H31	0.9300
C10—H10	0.9300	C23—H23	0.9300
C2—C4	1.374 (3)	C4—H4	0.9300
C2—C1	1.395 (3)	C29—C30	1.352 (4)
C2—H2	0.9300	C29—H29	0.9300
C1—C8	1.383 (3)	C32—H32	0.9300
C7—C5	1.377 (4)	C20—H20A	0.9600
C7—C8	1.380 (3)	C20—H20B	0.9600
C7—H7	0.9300	C20—H20C	0.9600
C22—C23	1.376 (4)	C30—H30	0.9300
			
C3—N2—N1	112.04 (16)	C17—N3—H3	119.1
C14—C9—C10	118.89 (18)	C27—C28—C29	122.0 (2)
C14—C9—C6	120.35 (18)	C27—C28—H28	119.0
C10—C9—C6	120.69 (18)	C29—C28—H28	119.0
C6—N1—C15	122.19 (16)	C16—N6—C18	123.7 (2)
C6—N1—N2	114.56 (15)	C16—N6—H6	118.1
C15—N1—N2	117.23 (15)	C18—N6—H6	118.1
C11—C12—C13	120.78 (19)	C24—C25—C26	120.5 (3)
C11—C12—Cl1	118.95 (17)	C24—C25—H25	119.7
C13—C12—Cl1	120.26 (18)	C26—C25—H25	119.7
C3—N4—C16	125.43 (17)	C21—C26—C25	120.3 (2)
C3—N4—N5	114.03 (15)	C21—C26—H26	119.8
C16—N4—N5	116.47 (16)	C25—C26—H26	119.8
C9—C14—C13	120.7 (2)	N6—C18—C20	110.5 (2)
C9—C14—H14	119.7	N6—C18—C27	109.80 (19)
C13—C14—H14	119.7	C20—C18—C27	112.6 (2)
C6—N5—N4	111.37 (16)	N6—C18—H18	107.9
N5—C6—N1	118.85 (17)	C20—C18—H18	107.9
N5—C6—C9	118.05 (18)	C27—C18—H18	107.9
N1—C6—C9	122.67 (16)	N3—C17—C19	109.74 (19)
N2—C3—N4	118.31 (17)	N3—C17—C21	111.32 (18)
N2—C3—C1	117.47 (18)	C19—C17—C21	112.34 (18)
N4—C3—C1	123.57 (18)	N3—C17—H17	107.7
O1—C15—N3	125.77 (19)	C19—C17—H17	107.7
O1—C15—N1	120.04 (18)	C21—C17—H17	107.7
N3—C15—N1	114.14 (18)	C17—C19—H19A	109.5
C12—C13—C14	119.5 (2)	C17—C19—H19B	109.5
C12—C13—H13	120.3	H19A—C19—H19B	109.5
C14—C13—H13	120.3	C17—C19—H19C	109.5
O2—C16—N6	126.6 (2)	H19A—C19—H19C	109.5
O2—C16—N4	120.1 (2)	H19B—C19—H19C	109.5
N6—C16—N4	113.33 (19)	C4—C5—C7	121.2 (2)
C12—C11—C10	119.2 (2)	C4—C5—Cl2	119.9 (2)
C12—C11—H11	120.4	C7—C5—Cl2	119.0 (2)
C10—C11—H11	120.4	C23—C24—C25	119.3 (3)
C26—C21—C22	118.0 (2)	C23—C24—H24	120.4
C26—C21—C17	122.4 (2)	C25—C24—H24	120.4
C22—C21—C17	119.5 (2)	C30—C31—C32	120.8 (3)
C11—C10—C9	121.0 (2)	C30—C31—H31	119.6
C11—C10—H10	119.5	C32—C31—H31	119.6
C9—C10—H10	119.5	C24—C23—C22	121.0 (3)
C4—C2—C1	120.4 (2)	C24—C23—H23	119.5
C4—C2—H2	119.8	C22—C23—H23	119.5
C1—C2—H2	119.8	C5—C4—C2	119.8 (2)
C8—C1—C2	118.7 (2)	C5—C4—H4	120.1
C8—C1—C3	122.51 (19)	C2—C4—H4	120.1
C2—C1—C3	118.25 (19)	C30—C29—C28	119.8 (3)
C5—C7—C8	119.0 (2)	C30—C29—H29	120.1
C5—C7—H7	120.5	C28—C29—H29	120.1
C8—C7—H7	120.5	C27—C32—C31	120.0 (3)
C23—C22—C21	120.8 (2)	C27—C32—H32	120.0
C23—C22—H22	119.6	C31—C32—H32	120.0
C21—C22—H22	119.6	C18—C20—H20A	109.5
C28—C27—C32	117.3 (2)	C18—C20—H20B	109.5
C28—C27—C18	121.4 (2)	H20A—C20—H20B	109.5
C32—C27—C18	121.3 (2)	C18—C20—H20C	109.5
C7—C8—C1	120.9 (2)	H20A—C20—H20C	109.5
C7—C8—H8	119.6	H20B—C20—H20C	109.5
C1—C8—H8	119.6	C31—C30—C29	120.0 (3)
C15—N3—C17	121.72 (18)	C31—C30—H30	120.0
C15—N3—H3	119.1	C29—C30—H30	120.0
			
C3—N2—N1—C6	−43.9 (2)	N2—C3—C1—C2	25.8 (3)
C3—N2—N1—C15	162.73 (18)	N4—C3—C1—C2	−163.65 (19)
C10—C9—C14—C13	−0.9 (3)	C26—C21—C22—C23	−1.9 (3)
C6—C9—C14—C13	−177.96 (19)	C17—C21—C22—C23	176.6 (2)
C3—N4—N5—C6	−44.5 (2)	C5—C7—C8—C1	0.1 (3)
C16—N4—N5—C6	156.90 (17)	C2—C1—C8—C7	−1.7 (3)
N4—N5—C6—N1	3.2 (2)	C3—C1—C8—C7	169.61 (19)
N4—N5—C6—C9	175.90 (16)	O1—C15—N3—C17	−7.2 (4)
C15—N1—C6—N5	−166.24 (18)	N1—C15—N3—C17	175.42 (19)
N2—N1—C6—N5	41.9 (2)	C32—C27—C28—C29	−0.3 (4)
C15—N1—C6—C9	21.4 (3)	C18—C27—C28—C29	179.0 (2)
N2—N1—C6—C9	−130.45 (18)	O2—C16—N6—C18	−1.1 (4)
C14—C9—C6—N5	50.0 (3)	N4—C16—N6—C18	−178.7 (2)
C10—C9—C6—N5	−127.1 (2)	C22—C21—C26—C25	0.7 (3)
C14—C9—C6—N1	−137.6 (2)	C17—C21—C26—C25	−177.9 (2)
C10—C9—C6—N1	45.3 (3)	C24—C25—C26—C21	1.1 (4)
N1—N2—C3—N4	2.3 (2)	C16—N6—C18—C20	124.9 (3)
N1—N2—C3—C1	173.40 (16)	C16—N6—C18—C27	−110.3 (3)
C16—N4—C3—N2	−161.03 (18)	C28—C27—C18—N6	−67.0 (3)
N5—N4—C3—N2	42.6 (2)	C32—C27—C18—N6	112.3 (3)
C16—N4—C3—C1	28.5 (3)	C28—C27—C18—C20	56.6 (3)
N5—N4—C3—C1	−127.88 (18)	C32—C27—C18—C20	−124.1 (3)
C6—N1—C15—O1	24.2 (3)	C15—N3—C17—C19	126.1 (2)
N2—N1—C15—O1	175.37 (18)	C15—N3—C17—C21	−108.9 (2)
C6—N1—C15—N3	−158.24 (18)	C26—C21—C17—N3	−36.0 (3)
N2—N1—C15—N3	−7.0 (3)	C22—C21—C17—N3	145.54 (19)
C11—C12—C13—C14	0.5 (3)	C26—C21—C17—C19	87.6 (2)
Cl1—C12—C13—C14	179.46 (16)	C22—C21—C17—C19	−90.9 (2)
C9—C14—C13—C12	0.4 (3)	C8—C7—C5—C4	1.4 (4)
C3—N4—C16—O2	27.1 (3)	C8—C7—C5—Cl2	−179.52 (17)
N5—N4—C16—O2	−177.10 (18)	C26—C25—C24—C23	−1.7 (4)
C3—N4—C16—N6	−155.15 (19)	C25—C24—C23—C22	0.4 (4)
N5—N4—C16—N6	0.7 (2)	C21—C22—C23—C24	1.5 (4)
C13—C12—C11—C10	−0.9 (3)	C7—C5—C4—C2	−1.4 (4)
Cl1—C12—C11—C10	−179.83 (17)	Cl2—C5—C4—C2	179.60 (18)
C12—C11—C10—C9	0.4 (3)	C1—C2—C4—C5	−0.3 (4)
C14—C9—C10—C11	0.5 (3)	C27—C28—C29—C30	−0.1 (4)
C6—C9—C10—C11	177.59 (19)	C28—C27—C32—C31	0.1 (4)
C4—C2—C1—C8	1.7 (3)	C18—C27—C32—C31	−179.2 (2)
C4—C2—C1—C3	−169.9 (2)	C30—C31—C32—C27	0.4 (4)
N2—C3—C1—C8	−145.6 (2)	C32—C31—C30—C29	−0.9 (5)
N4—C3—C1—C8	25.0 (3)	C28—C29—C30—C31	0.7 (4)

### Hydrogen-bond geometry (Å, º)

**Table:**

*D*—H···*A*	*D*—H	H···*A*	*D*···*A*	*D*—H···*A*
N3—H3···N2	0.86	2.21	2.613 (2)	109
N6—H6···N5	0.86	2.13	2.573 (2)	112
